# Nutrition management programme for older adults in Ningxia nursing homes, China: evidence-based practice and the Delphi method

**DOI:** 10.1017/S1368980024002453

**Published:** 2024-12-02

**Authors:** Haiyan Liu, Li Chen, Yanhua Ning, Yahong Guo, Weijuan Kong, Xiongxiong Lv, Meiman Li, Ting Jiang

**Affiliations:** 1 School of Nursing, Ningxia Medical University, Yinchuan, People’s Republic of China; 2 Wuzhong People’s Hospital, Wuzhong, People’s Republic of China; 3 School of Nursing, Gansu University of Chinese Medicine, Lanzhou, People’s Republic of China

**Keywords:** Evidence-based practice, Nursing home, Nutrition, Older adults

## Abstract

**Objectives:**

To construct an evidence-based practice programme for the nutrition management of older adults in nursing homes. The programme will provide a basis for improving or solving the nutrition management problems of older adults in nursing homes.

**Design:**

The study is based on guideline evidence and Delphi method. The evidence was comprehensively searched, assessed and summarized, and the best evidence and a preliminary programme for nutrition management of older adults in nursing homes were aggregated. Then, the Delphi method was used to assess the applicability of the preliminary programme and the obstacle factors to modify, supplement and improve the nutrition management programme.

**Setting:**

Baseline survey data were collected from three nursing homes in Ningxia, China, and guideline evidence was obtained through systematic searches of the Cochrane Library, PubMed and other scientific databases, as well as relevant official websites.

**Participants:**

A total of 350 older adults residing nursing homes and 160 nurses participated in the baseline survey. To ensure the programme’s applicability and identify potential implementation obstacles, fifteen experts from local grade A hospitals, nursing homes and community health centres were consulted for review.

**Results:**

A fourteen-item, fifty-six-best-evidence nutrition management programme for older adults in nursing homes was developed based on five guideline evidences and baseline survey findings.

**Conclusions:**

This is a systematic and comprehensive nutritional management programme for older adults in nursing homes based on guideline evidence, which can provide a standardised basis for the implementation of scientific nutritional management in nursing homes in Ningxia. Managers should promote the translation of evidence into practice in accordance with the specific circumstances of individual nursing homes.

Older adults are susceptible to malnutrition as a consequence of age-related physiological decline, restricted access to nutritionally dense food and the presence of multiple chronic illnesses. Malnutrition is a highly prevalent condition in older adults, imposing a significant burden on health, social and aged care systems^(1)^. Malnutrition increases the risk of adverse clinical outcomes in older adults, including frailty, osteoporosis, muscle wasting and mortality^(1)^. Consequently, the number of inpatients and treatment costs rise, resulting in a substantial economic burden on families and society^(2)^.

China is among the most rapidly ageing countries in the world, with the largest ageing population. As the long-term care needs of older adults increase, nursing homes are proving to be an effective support structure for the care business in various countries^(3,4)^. In excess of two million senior citizens elect to reside in nursing homes throughout China^(5)^. These facilities accommodate a significant number of older adults with disabilities or semi-disabilities and frail health, as well as individuals with a combination of chronic diseases^(4)^. Nursing home patients may be at a heightened risk of nutritional vulnerability. The prevalence of malnutrition among nursing home residents in different regions of China ranged from 14·4 to 52·4 %^(6–9)^. The prevalence of nutritional issues among older adults may be higher in Northwest China, particularly in Ningxia^(10)^.

Currently, there are a number of evidence-based strategies that can be employed in the management of malnutrition in older adults^(11)^. However, there is a paucity of reports on science-based nutrition management methods for older adults in nursing homes^(12)^. The incorporation of nutritional guidelines is inadequate, and low-value care is commonplace. There is a lack of standardisation and personalised nursing intervention measures plans^(13)^, which results in a significant discrepancy between the interventions that are known to be effective and the care that is applied^(1)^. In China, there is a dearth of nutrition professionals in pension institutions, and the nutrition management is not standardised. Furthermore, a reasonable and effective nutrition management model has yet to be established^(14)^. The situation is particularly acute in Ningxia. Consequently, the development of a set of nutrition management programme suitable for older adults in Ningxia nursing homes represents a crucial avenue for advancing the scientific management of older adults’ care institutions.

Evidence-based nursing represents a significant and valuable nursing methodology. It offers the potential to provide more effective evidence-based support based on evidence-based evidence for the nursing object, thereby facilitating improvements in nursing effectiveness^(15)^. To enhance the integration of evidence-based nutritional knowledge into clinical practice, guidelines must be accompanied by active implementation strategies that are informed by local contextual factors (barriers and facilitators) influencing the delivery of care^(1)^. In conclusion, the study, based on the baseline survey, indicates that it is essential to utilise the existing guidelines and Delphi method^(16)^ method to assess the applicability of these recommendations and analyse the obstacle factors. This will facilitate the development of a more appropriate nutrition management programme for nursing homes, bridge the gap between evidence and practice and provide a robust foundation for the implementation of scientific nutrition management for older adults in nursing homes.

## Methods

The JBI evidence-based healthcare model was employed as a theoretical framework for the study development process^(17)^. This entailed a baseline survey of nursing homes to identify and establish evidence-based nursing problems, a systematic retrieval of diet, nutrition, and dietary guidelines evidence for older adults according to the problems, and an application of AGREE II^(18)^ to evaluate the quality of evidence. The resulting data informed the formulation of the initial draft of a nutrition management plan for older adults in nursing homes. The expert team employed the Delphi method to classify the suggestions, which were then modified, supplemented and perfected to form the final programme.

### Study team

The study team was comprised of an evidence-based nursing tutor with a background in nursing, nutrition and food hygiene, as well as seven postgraduate students who had completed the requisite evidence-based nursing course examinations. The team had received training in diet care for older adults, diet and nutrition knowledge and food intake estimation. In addition to these professionals, the evidence-based nursing research involved the participation of an evidence-based medicine teacher, a nursing home manager, a nutritionist and a nursing home manager. The tutor bears responsibility for the comprehensive planning, guidance and supervision of the construction of the management programme. The inclusion of nursing home managers can help the group fully explore the gap between theory and practice in nutrition management from a leader’s perspective. Four postgraduate students were responsible for conducting the baseline survey, two postgraduate students were responsible for conducting field observations and four postgraduate students were responsible for evidence retrieval, quality evaluation, evidence collection and expert correspondence data sorting and analysis. In the event of any issues or disagreements, two evidence-based teachers were consulted to make a decision. Additionally, sixteen experts in geriatric care, nutrition management and community health services were selected to form the expert consultation team.

### Baseline survey

Following the completion of the literature review and the study group meeting, we proceeded to identify evidence-based nursing issues affecting older adults, nurses and the nursing home management model. From August to December 2020, we conducted a cross-sectional study, recruiting 350 older adults and 160 nurses from three nursing homes for a baseline survey. The objective was to identify current problems with nutritional management in nursing homes and to utilise the findings as part of the evidence base for nutritional management programmes. The study included all older adults aged 60 years and over who had been living in a nursing home for at least 1 year in order to gain an insight into the nutritional status and problems of older adults in nursing homes; older adults in poor physical condition and unable to communicate effectively were excluded. All nurses who had worked continuously for 1 year were included.

A general data questionnaire was designed for the purpose of collecting information on a range of factors pertinent to the lives of older adults, including age, sex, occupation, diet and living habits, nutritional knowledge and attitude. The Dietary Self-Efficacy Scale (DSE)^(19,20)^ was employed to evaluate the self-efficacy of older adults in four domains: diet, healthy eating skills, eating behaviour control and weight control in social situations. The Short Form Mini Nutritional Assessment (MNA-SF)^(21)^ was employed for the purpose of evaluating the nutritional status of the older adult population. The Simple FFQ (FFQ25)^(22)^ was employed to ascertain the participants’ dietary intake. Additionally, nurses’ attitudes towards nutritional care were gauged through administration of the Staff Attitudes to Nutritional Nursing Care Geriatric scale (SANN-G)^(23)^. To gain further understanding, two postgraduate students conducted 2-month field observations^(24)^, noting the feeding style, food types, food arrangement and individual meal preparation. These observations were promptly documented within 24 h.

### Searching for guideline evidence

A search strategy based on keywords and Mesh terms was formulated according to the characteristics of various databases and websites. This was followed by a systematic search was performed in PubMed, Embase, Web of Science, China Biomedical Literature Database (CBM), China National Knowledge Infrastructure (CNKI), WANGFANG DATA and VIP Database. Furthermore, seven additional online sources were consulted, including the Cochrane Library, the National Guidelines Library (NGC), International Guidelines Consortium (GIN), the European Society for Parenteral and Enteral Nutrition (ESPEN), the UpToDate platform, the Medline Live Guide Net and the Chinese Nutrition Society were also been searched.

The target population comprised older adults residing in nursing homes. The evidence base comprised guidelines, presented in either Chinese or English. To gain a comprehensive insight into the study’s key findings, all available guidelines published within the last 10 years were considered, with the exception of those that were directly translated, updated and republished guidelines.

**Figure 1.**
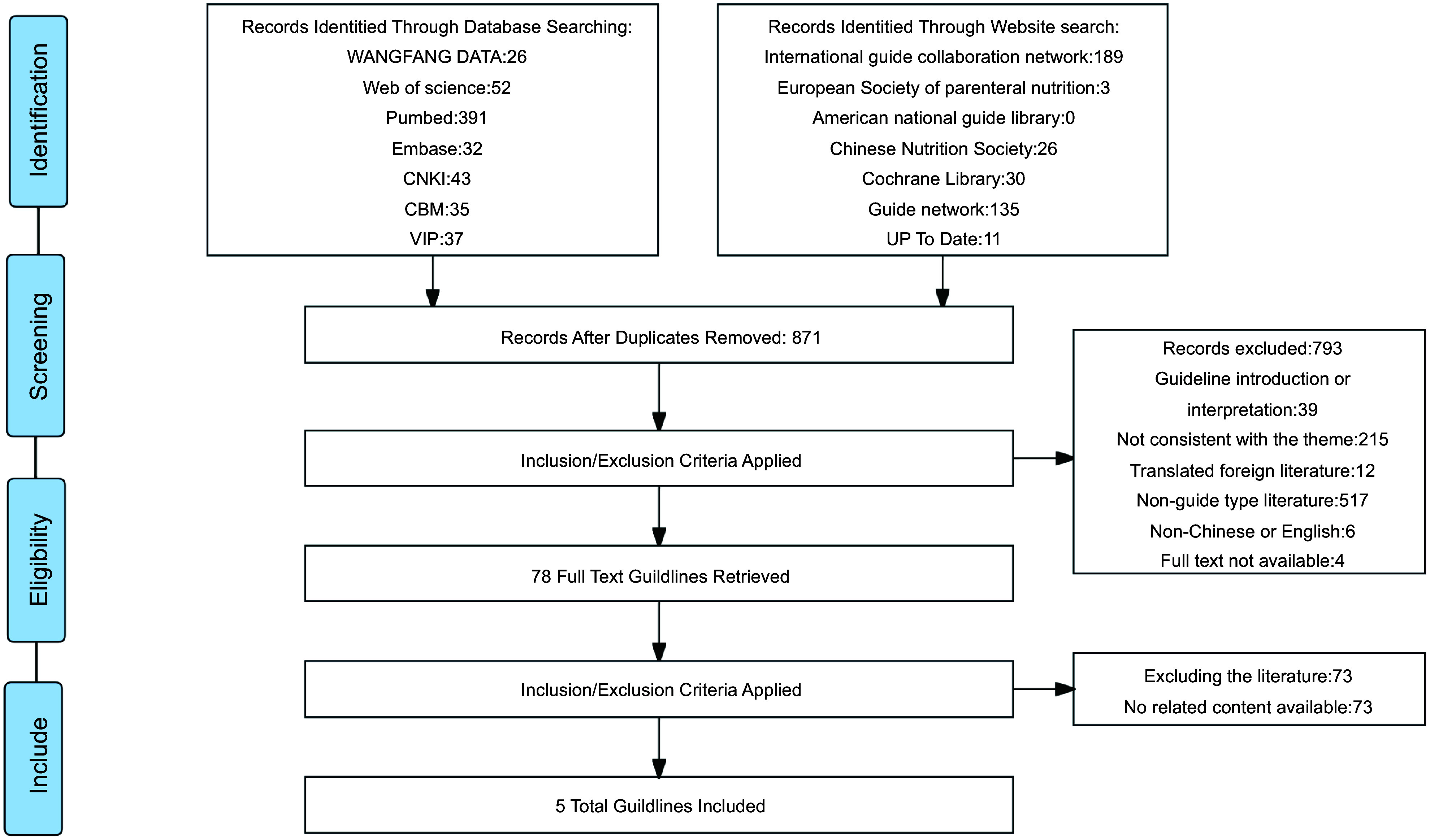
PRISMA flow diagram of search and selection process.

All retrieved publications were imported into the Endnote X9 software for screening according to the established inclusion criteria. Two postgraduate students independently screened the evidence according to the criteria. Following the removal of duplicate guidelines using the software, irrelevant guidelines were excluded based on a review of the title and abstract. Irrelevant guidelines were also excluded through a second review of the full text. All discrepancies were discussed or consulted with a third investigator until a consensus was reached. The PRISMA flow diagram of the search and selection process is shown in Fig. 1.

### Quality assessment of the guideline evidence

Two postgraduate students employed the AGREE II instrument^(18)^ to assess the included guidelines. The evaluation system comprises six domains, namely scope and purpose, stakeholder involvement, rigour of development, clarity of presentation, applicability and editorial independence. A total of twenty-three items are included in the system. The guideline is classified according to the percentages of standardised items in the six domains and the score of each item. Guidelines with a score >60 % in all six domains are classified as high quality and can be directly recommended without modification (grade A). Guidelines with a score of 60–80 % in all domains or 60–79 % in five domains are classified as moderate quality and can be recommended with modifications (grade B). Finally, guidelines with a score of >80 % in only two domains or a score of 80–89 % in only three domains are classified as low quality and should be recommended with significant revisions (grade C). Those score >60 % in only two domains were deemed to possess moderate quality and thus were recommended for revision and improvement (B-recommended). Those scoring >60 % in only two domains were considered to possess low quality, while those scoring in only one domain were deemed to possess very low quality (C-not recommended). Furthermore, the intraclass correlation coefficient (ICC) was employed to assess the consistency of the assessment results. An ICC value of >0·75 was interpreted as indicative of a high degree of consistency among the experts^(25)^.

### Extraction and synthesis of guideline evidence

Two postgraduate students conducted preliminary screening of the recommendations included in the guideline independently. They then and extracted the recommended items involving nutrition, diet and diet for older adults into the literature information table. In the event of discrepancies between the recommendations, the recommended items in the guidelines will be extracted anew until the extracted recommended items are consistent. In the event of continued disagreement, the evidence-based nursing teacher was asked to provide a ruling. The recommendations deemed suitable for implementation in nursing homes based on the baseline survey of nursing homes should be identified and any amendments to the recommended items that are not suitable for implementation should be proposed. Following the collation of all items, a decision will be made as to whether they meet the requisite criteria or whether they should be retained following modification.

The JBI Evidence-Based Health Care Center’s Evidence Grading Approach and Recommendation Grading System (2010) was employed to grade the included evidence and formulate recommendations^(17)^. When there is a conflict of opinion between different sources of evidence, inclusion principle is followed to prioritise high-quality evidence and national guidelines, in alignment with the purpose of this study.

In accordance with the recommendations set forth in the included guideline evidence, baseline survey and field observations data from nursing homes, we have summarised and determined the level of evidence and strength of recommendation in order to construct the most comprehensive set of evidence for nutrition management in nursing homes and draft a nutrition management programme information booklet for older adults in nursing homes.

### Applicability and obstacles of guideline evidence

The information booklet was distributed to the sixteen experts via email as a consultation questionnaire. The objective was to ascertain their views on the obstacles to the implementation in nursing homes. Experts are selected based on their experience, with the exception of nursing home nurses and managers, who are required to possess a bachelor’s degree or higher, have completed a minimum of 5 years of service and hold an intermediate or senior title. The initial phase encompassed the following elements: (a) basic information about the experts and (b) an evaluation of the suitability of each component of the nutrition management programme and an analysis of obstacles of the implements to its implementation. A five-point Likert scale was employed to assess the suitability of the items: (c) expert authority: the basis for the expert’s judgement and familiarity with the consulting content and (d) expert opinions and recommendations pertaining to this study.

Two postgraduates students were responsible for organising and synthesising the opinions and suggestions provided by experts. Following the evidence-based group discussion, the content requiring revision and deletion was identified. Subsequently, the initially formulated nutrition management programme underwent further revision and improvement. Following modifications to the programme in accordance with the results of the initial round of consultation, a second round of expert consultation with experts was conducted after a 2-week interval. Additionally, the consultation opinions and modifications results were supplemented. Following two rounds of consultation, the experts’ opinions demonstrated a notable degree of consistency. Two postgraduate students collated and synthesised all comments and suggestions and subsequently identified the requisite modifications and deletions through an evidence-based group meeting.

The response rate of the questionnaire is indicative of the enthusiasm of experts in this study. In order to assess the authority of experts, the authority coefficient (*Cr*)^(26)^ was employed, with the calculation formula being as follows: *Cr* = (*Ca* + *Cs*)/2. The expert’s judgement basis for determining the suitability of the content of the nutrition management programme includes theoretical knowledge, practical experience, reference to data and the subjective feeling of experts. The degree of *Ca* was categorised into three levels from low to high in order to facilitate analysis. The degree of experts’ familiarity that experts have with the programme (*Cs*) is rated on a five-point scale, with lower numbers indicating a lesser degree of familiarity and higher numbers indicating a greater degree of familiarity. The coordination of expert opinions was quantified by the Kendall coefficient of concordance (*W*) and CV. A higher the *W* value indicates superior coordination. A smaller the *CV* value indicates a higher degree of coordination among expert opinions. A *CV* value of ≤ 0·25 is generally considered acceptable. The degree of concentration of expert opinions is described by the average of applicability score. A higher average indicates a more concentrated the expert opinion and a higher-importance indicator. The content of the applicability score includes the representativeness of the content of each item, the importance of the item and the feasibility of the evaluation. Items and content were screened using a score of > 3·5 and CV of < 0·25.

## Results

### Baseline results

**Figure 2.**
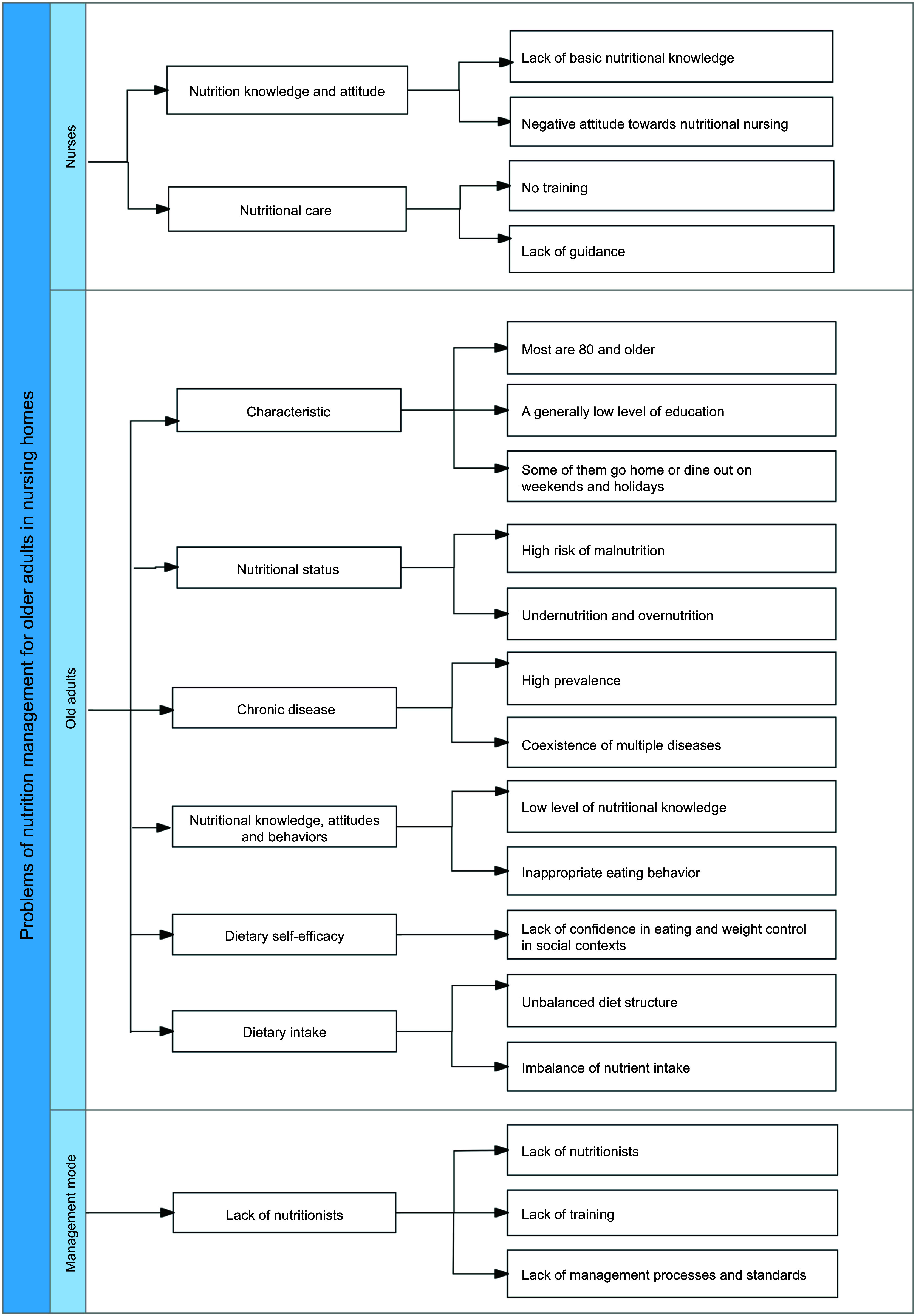
Nutrition management problems of older adults in nursing homes.

Based on the findings of the baseline survey and field observations of older adults and nurses in the nursing homes, as well as the on-site meeting discussions of the study team, three aspects of the problem were identified from the perspectives of the older adults, the nurses and the management model. These findings are illustrated in Fig. 2.

### Included evidence

The study encompassed a total of five guidelines, as detailed in Table 1. Of these, three have been published by the European Society of Clinical Nutrition and Metabolism, one by the Chinese Medical Association Parenteral and Enteral Nutrition Branch and one has been published in the Journal of Nutrition Health & Aging. Four of the guidelines are in English and one is in Chinese.

**Table 1.** Information on the included guidelines for the nutrition management of older adults

No.	Guideline	Publication year	Issuing agency
1	Guidelines for the application of parenteral and enteral nutrition in elderly patients in China (2020)^(27)^	2020	Geriatric Nutrition Support Group, Parenteral and Enteral Nutrition Society, Chinese Medical Association
2	ESPEN guideline on clinical nutrition and hydration in geriatrics^(11)^	2019	European Society for Clinical Nutrition and Metabolism
3	ESPEN guidelines on nutrition in dementia^(28)^	2015	European Society for Clinical Nutrition and Metabolism
4	Nutritional guidelines for older people in Finland^(29)^	2014	Journal of Nutrition Health & Aging
5	Clinical practice guidelines from the French health high authority: Nutritional support strategy in protein-energy malnutrition in the elderly^(30)^	2011	European Society for Clinical Nutrition and Metabolism

ESPEN, European Society for Parenteral and Enteral Nutrition.

The AGREE II evaluation results indicated that five guidelines were recommended with modifications (Level B). The ICC values for the two reviewers’ evaluation consistency test for the five guidelines were all > 0·75, indicating a high degree of coordination of expert opinions and relatively consistent opinions (Table 2). The evidence was summarised in five aspects: management team, nutrition screening, nutrition management, classified management and nutrition monitoring and effectiveness evaluation. This information was organised into an information booklet (Appendix 1). The results of the baseline survey were presented as G-level evidence (practice views or expert consensus).

**Table 2.** Results of quality assessment and consistency test of the guidelines

Column	Quality assessment^a^						Recommendation grade	ICC
No.	Domain 1	Domain 2	Domain 3	Domain 4	Domain 5	Domain 6		
1	72	36	55	92	79	54	B	0·853^*^
2	89	56	79	86	83	50	B	0·930^*^
3	83	58	74	83	79	54	B	0·754^*^
4	83	64	56	78	75	29	B	0·778^*^
5	86	47	64	78	85	0	B	0·816^*^

ICC, intraclass correlation coefficient.

a The scores of the six domains of AGREE II (scope and purpose, stakeholder involvement, rigour of development, clarity of presentation, applicability and editorial independence).

* The ICC *P*-value is significant at the 0·001 level.

### Applicability evaluation and obstacle factors review

Following two rounds of consultation, we received responses from fifteen experts (one expert did not complete the final reply due to failing health). The general information of the experts is presented in Appendix 2. The experts possess a considerable degree of authority, as evidenced by the first and second rounds of consultation, with a Cronbach’s α of 0·84 and 0·85, respectively. They have expressed positive sentiments regarding the two rounds of correspondence in this study, particularly with regard to the effective recovery rates. The response rates for the questionnaires were 93·8 % and 100 %, respectively, and the degree of coordination of opinions was also satisfactory, with a Kendall coefficient of concordance (*W*) of 0·376 and 0·379, respectively. With the exception of the ‘applicability of the rehabilitation therapists and person in charge’, the mean which applicability scores of each item in the two rounds were 3·27–4·80 and 4·0–5·0, respectively. Moreover, the concentration of expert opinions in the second round of correspondence was higher.

The average applicability score was set at >3·50, and the CV was set at <0·25, which were used as the criteria for screening items and content^(31)^. Based on feedback from experts and on-site meetings of the group team, modifications and improvements were made to the nutrition management programme. The items and contents modified and refined by the expert consultation are listed in Appendix 3. Finally, four dimensions of obstacles were identified in the implementation of the nutrition management programme, namely personnel, materials, systems and management (Table 3).

**Table 3.** Obstacles in the implementation of nutrition management programme in nursing homes

Dimension	Obstacle factors
People	(a) The level of staffing of professional nurses and dietician. (b) Nurses and geriatric carers with low education level, acceptability of nutrition knowledge and proficiency in the use of nutrition screening assessment tools. (c) The degree of professionalism of nutritionists in the dietary nutrition matching of older adults. (d) Physician’s ability to manage adverse effects of nutritional intervention regimens. (e) The proficiency of the catering staff in the preparation of nutritious meals. (f) Employees’ job responsibilities and contents. (g) Workload issues in nutritional management implementation. (h) The degree of cooperation of the older adults and their family members, the ability to accept nutrition knowledge and the ability to pay for nutrition management costs.
Materials	(a) Introduction of nutritional screening assessment tool and its proper and effective use. (b) Accurate collection and preservation of data on nutrition assessment, dietary assessment and dietary habits of older adults.
Systems	(a) Continuity and standardisation of nutrition management process. (b) Lack of nutrition monitoring and emergency treatment mechanism for nutrition-related complications. (c) Nutrition care training and assessment systems are incomplete.
Management	Encouragement and supervision mechanisms are not perfect.

**Figure 3.**
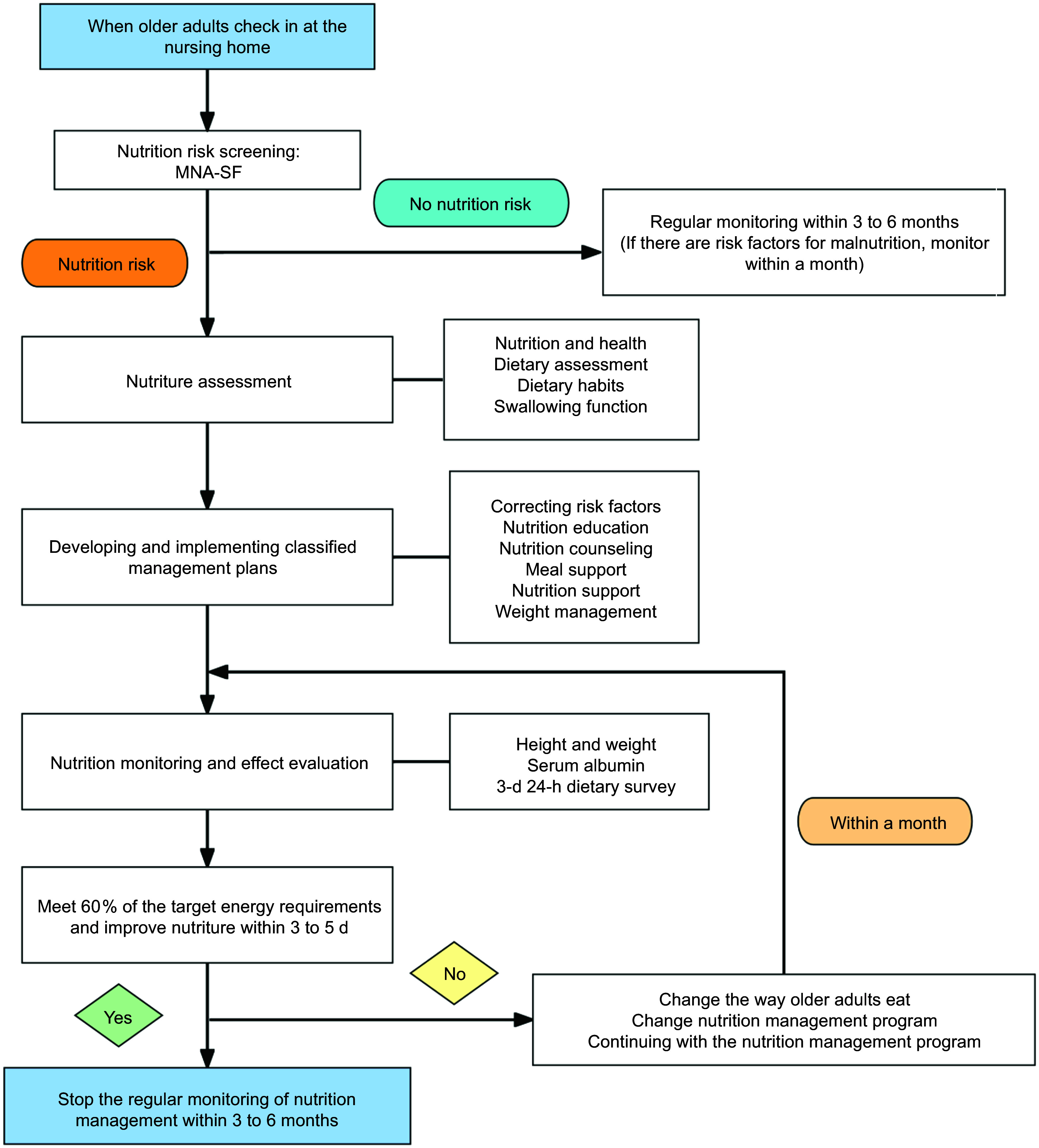
Nutrition management process for older adults in nursing homes. MNA-SF, The Short-Form Mini-Nutritional Assessment.

Following a series of modifications and enhancements, we have at last devised a comprehensive programme for the nutrition management of older adults in nursing homes. The programme comprises three aspects: management team, management objects and management content. The latter includes nutrition screening, nutrition assessment, formulation and implementation of a classification management, and nutrition monitoring and effect evaluation in four areas, fourteen items and fifty-six contents (Appendix 4). To enhance efficiency and convenience, a nutrition management process for older adults in nursing homes has been developed (Fig. 3).

## Discussion

Nutrition problems among older adults residing in nursing homes are a significant challenge. Nutrition intervention represents a crucial strategy for enhancing clinical outcomes in older adults. Currently, nursing homes lack a comprehensive and scientific nutrition management system, as well as a synthesis of expert opinion and evidence-based guidance on specialised nutrition management. There is a need for a unified action guideline to address these shortcomings^(30)^. The identification and utilisation of evidence-based methods to identify and treat older adults at risk of or with malnutrition are essential to improve nutrition and health outcomes, including quality of life^(32)^. Therefore, it is significant for us to combine the actual situation of nursing homes and adopt evidence-based nursing practice to summarise the evidence to construct a scientific nutrition management programme for older adults in nursing homes. We included guideline evidence of a high level of recommendation to construct the programme, ensuring its scientific rigour.

The application of evidence is bound to encounter obstacles at various levels^(33)^. It is thus imperative to assess the obstacles to evidence application and develop targeted action strategies. This study also sought input from experts in relevant fields to evaluate the suitability of the nutrition management programme using the Delphi method and analyse the barriers to implementation. They proffered corresponding revision opinions and solutions in terms of management personnel, responsibilities, content and so forth, combining their rich work experience and the clinical situation of older adults care institutions.

In the construction of this programme, consideration was given to a number of objective conditions, including the baseline background of nursing homes, the management system and the human resources available. Given the distinctive characteristics of the nursing home population, we adjusted the content of the programme was tailored based on the experience and suggestions of experts in the field. In regard to the ‘5 + 2’ elderly care model (which encompasses five workdays a week and a hosting model for normal social activities with peers, as well as 2 d on weekends designated as family pensions, during which older adults gather share the joy of family)^(32)^, we have incorporated the perspectives of family members of older adults into the formulation of management strategies. Furthermore, in view of the dearth nutritionists in nursing homes and the coexistence of multiple diseases among older adults, we have established several avenues for collaborative management between nursing homes and medical institutions, including face-to-face and WeChat consultation. The primary challenge to implementing the programme is the lack of nutritional knowledge and expertise among members of the nursing home management team. To address this, the programme places a particular emphasis on nutrition, nursing education and skills training at the personnel level. These aspects ensure the feasibility of the management programme.

The ‘Integrated Eldercare Services with Medical Care’ model represents an innovative approach to elderly care that integrates medical care and elderly care services^(34)^. This model has the potential to enhance the capacity of elderly care and medical institutions, optimise the service industry and promote healthy ageing. It also provides a robust foundation for the implementation of effective nutrition management programmes in nursing homes^(32)^. In light of these considerations, we present the following recommendations to ensure the successful implementation of nutrition management programme in nursing homes:

1. The effective implementation of nutrition management programme is contingent upon the involvement of professional nurses. The cultivation of a professional nutrition specialist team represents a crucial strategy for enhancing the efficacy of nutrition management. As the organiser and coordinator of project implementation, it is incumbent upon managers to harness the enthusiasm and capabilities of nurses. It is imperative that the functions of nutrition specialist nurses are not overlooked. They require comprehensive training in nutrition management programmes and processes. Additionally, they must be equipped with the knowledge to utilise nutritional screening tools, which are an integral part of routine care records. The long-term mechanism of nutrition management must be integrated into the existing framework of older adult nursing work systems. Furthermore, there is a pressing need to enhance the training and assessment mechanisms for nutrition nursing within the current evaluation system.
2. China advocates a medical care combination model of cooperation between nursing institutions and communities or hospitals and should provide outpatient or telephone consultation services for older adults in nursing homes. It is also incumbent upon the government to prioritise the building and stabilisation of a robust talent pool for senior care services, such as the provision of incentives and subsidies for graduates and senior care workers alike. Furthermore, the government also ought to invest more funds and resources to encourage the development of low-income nursing homes^(35)^.

China’s National Nutrition Plan (2017–2030) underscores the significance of nutrition enhancement for older adults and the associated implementation requirements. Older adults residing in nursing homes constitute a distinct social group, and nutrition has consistently been a pivotal area of concern. It is imperative that managers implement a standardised and applicable nutrition management programme for this group. This will facilitate the enhancement of nurses’ nutrition nursing skills, the advancement of nutrition improvement actions for older adults and the optimisation of home management. China is implementing the Integrated Eldercare Services with Medical Care model. The implementation of effective policies can significantly contribute to the development of comprehensive elderly care services, and medical care elderly care models develop significantly.

## Conclusions

This study employed evidence-based nursing methods to address the current challenges in the nutrition management of older adults in Ningxia nursing homes. It utilised guideline evidence to elucidate the impediments to evidence implementation and to identify coping strategies through expert consultation, thereby completing the practical application programme of the optimal guideline evidence. The content of the nutrition management programme constructed in this study is comprehensive. It is our contention that the promotion and application of this programme will result in the implementation of a systematic and standardised nutrition management programme for older adults. Although this method is scientifically sound, the recommendation level of some evidence is somewhat low. Consequently, we need to conduct an empirical study of this programme to verify its feasibility and efficacy in order to achieve the ultimate goal of improving the malnutrition of older adults in nursing homes. However, the baseline survey was conducted in only three nursing homes, which may not fully reflect the current status and problems of nutritional management in nursing homes in Ningxia, thus affecting the generalisability of the findings.

## Supporting information

Liu et al. supplementary material Liu et al. supplementary material

## Supplementary material

For supplementary material accompanying this paper visit https://doi.org/10.1017/S1368980024002453
