# Supplementary material for: Nutrition management programme for older adults in Ningxia nursing homes, China: evidence-based practice and the Delphi method
Source: Public Health Nutr. 2024 Dec 2;27(1):e262. doi: 10.1017/S1368980024002453 (PMC11705011; doi:10.1017/S1368980024002453)
Supplement: Liu et al. supplementary material [file S1368980024002453sup001.pdf]

Appendix 1 Information booklet for nutrition management guideline evidence of older adults in nursing homes

Item	Contents of evidence	Evidence grade	Recommendation grade
Management team	Multi-mode and multi-disciplinary teams.	III	A
Nutrition screening	1.Screening for nutritional risk is recommended for all older adults.	IV	A
	2.The MNA-SF is recommended as a nutritional screening tool for older adults.	II	A
	3.Screening for the first time when older adults are admitted to a nursing home and monthly thereafter.	IV	A
	4.Determinants of screening frequency: clinical status and level of risk.	IV	A
Nutrition assessment	1. Assessment of disease severity, dietary intake, laboratory tests, weight and body composition measurements, etc.	IV	A
	2. Assessment of diet quality, dietary diversity, and intake of food, energy, and nutrients.	IV	A
Classification management	**1. Eliminate the risk factors for malnutrition**		
	(1) The underlying causes of malnutrition should be identified and eliminated as much as possible.	IV	A
	(2) Unnecessary dietary restrictions should be avoided.	IV	A
	(3) Provide technical or human assistance with meals, oral and dental care, assessment of medication and diet, management of underlying diseases.	IV	A
	**2. Nutrition education**		
	**(1)** People who need nutrition education		
	1) Provide nutrition information and education to older persons who are malnourished or at nutritional risk.	III	A
	2) Provide nutrition education to health professionals and caregivers.	III	A
	3) Provide nutrition education to family members of older adults and cafeteria staff.	G	C
	**(2) Contents of nutrition education**		
	1) Older adults: basic knowledge of nutrition and disease-related, weight control and healthy eating skills.<br>2) Nurses: knowledge of elderly nutrition care and disease-related nutrition; knowledge of elderly nutrition guidelines and individualized nutrition needs knowledge.<br>3) Family members of older adults: basic nutrition knowledge such as dietary guidance for older adults at home during holidays and weekends, recommended daily intake of various foods, and nutrient food sources.	G	C

Appendix 1 (Continued)

Item	Contents of evidence	Evidence grade	Recommendation grade
Classification management	4) Cafeteria staff: older adults basic nutrition knowledge, food nutrition collocation and specialized food production training.	G	C
	**(3) Forms of nutrition education**		
	1) Distributing nutrition manuals, giving nutrition knowledge lectures, setting up nutrition publicity boards, and conducting public nutrition counseling.	G	C
	2) Nutrition and nursing care-related education.		
	3) Nutrition and nursing care-related education.		
	**(4) Cautions**		
	Different educational methods and contents are selected according to the educational level and nutrition knowledge level of the educational objects.	G	C
	**3. Nutrition counseling**		
	**(1)** Individual nutrition counseling		
	**(2)** Provide individualized nutrition counseling to seniors and/or caregivers.		
	1) Provide individualized nutrition counseling to seniors and carers.	III	A
	2) Professional dietitians provide individualized nutrition counseling, including face-to-face and telephone follow-up.	IV	A
	**4. Meal support**		
	**(1)Meal assistance:**		
	1) Provide meal assistance to older adults who are at risk of malnutrition or nutrition and have food dependency.	I	A
	2) Provide meal delivery service for some elderly people according to their physical activity.	G	C
	3) Design menus according to the preferences of older adults, adjust the texture and concentration of food according to their chewing and swallowing ability.	IV	A
	4) Increase the supply of vegetables, fruits, milk, fish, shrimp and nuts to provide a varied diet.	G	C
	**(3) Special meal support**		
	1) Establish a diet supply area for patients with diabetes and hypertension, and formulate diet packages for patients with diabetes and hypertension for patients according to the patients' conditions.<br>2) Provide low-fat diet for older adults with obesity, suffering from cardiovascular and cerebrovascular diseases.	G	C

Appendix 1 (Continued)

Item	Contents of evidence	Evidence grade	Recommendation grade
Classification management	**(4) Environmental support**		
	1) Provide a pleasant, comfortable eating environment.	I	A
	2) Encourage older adults to eat together with others .	IV	A
	**(5) Meal frequency and time**		
	1) Eat at least three meals a day, and snacks are recommended to increase meal frequency.	IV	A
	2) Eat on time and provide sufficient meal time.	IV	A
	**5. Nutritional support**		
	**(1) Goals of nutritional support**		
	1) Achieve an energy intake of 20–30 kcal/(kg-d).	II	B
	2) Protein intake of 1.0–1.5g/ (kg-d).	III	B
	3) Individualized adjustment based on nutrition, physical activity level, disease status, and tolerance of older adults.	III	A
	**(2) Fortified foods**		
	In addition to a targeted diet, fortified foods should be provided to older adults who are malnourished or atnutritional risk.	III	A
	**(3) Oral nutritional supplements (ONS)**		
	1) When dietary counseling and food fortification are insufficient to increase dietary intake and achieve nutritional goals, ONS should be provided to older adults who are undernourished or at nutritional risk.	IV	A
	2) According to the taste and eating ability of older adults, choose the type, taste and texture of the food, and determine the time to eat.	IV	A
	3) ONS provides 400–600 kcal/(kg-d) with 30g of protein.	I	A
	4) The effect of the ONS should be evaluated monthly.	IV	A
	5) Attention should be paid to adjusting the taste of the preparation taste and psychological care measures to improve the compliance of older adults.	III	A
	6) Combination of diet with ONS improved nutrition in older adults, but did not affect dietary intake.	I	A
	**(4) Enteral Nutrition (EN)**		
	1) Older adults at risk of malnutrition or malnutrition and with normal or basically normal gastrointestinal function should choose EN and make a reasonable EN plan according to their characteristics.	II	A
	2) If an EN is needed, start it up immediately.	IV	A
	3) Using standard whole protein formula and optimizing fatty acids can improve lipid metabolism and reduce the occurrence of cardiovascular events.	III	A

Appendix 1 (Continued)

Item	Contents of evidence	Evidence grade	Recommendation grade
Classification management	4) Fiber intake of 25 g/d helps to reduce constipation and improve clinical outcomes in tube-fed patients.	II	A
	5) Evaluate the effect and tolerability of EN and periodically reassess periodically as clinical circumstances change.	IV	A
	**6. Weight management**		
	(1) Overweight: avoid dieting; weight loss recommendations should vary from person to person and pay attention should be paid to assessing the impact of excess weight on quality of life.	IV	A
	(1) Obese: moderate energy restriction and slow weight loss; dietary intervention should be combined with physical activity to preserve muscle mass.	IV	A
Nutrition monitoring and effect evaluation	1. Anthropometric indicators: body weight.	IV	A
	2. Reassessment of the nutrition.<br>(1) MNA-SF.	II	A
	(2) Food intake: food intake 24 hours a day for 3 days. The frequency of monitoring was determined based on the clinical status, the severity of malnutrition, and the weight changes.	IV	A
	3. Serum albumin: serum albumin measurement is recommended to evaluate the effect of nutritional management.	IV	A

Appendix 2 General information of the experts

Item		Number	Proportion(%)
Gender	Male	4	26.7
	Female	11	73.3
Age	25-	3	20.0
	35-	7	46.7
	45-	5	33.3
Educational background	Bachelor's degree	5	33.0
	Master's degree	9	60.0
	Doctoral degree	1	6.7
Professional title	Senior	4	26.7
	Deputy senior	3	20.0
	Intermediate	4	26.7
	Other	4	26.7
Professional field	Aged care and management	6	40.0
	Nutrition management	4	26.7
	Community health service	5	33.3
Working years	5-	6	40.0
	10-	6	40.0
	20-	3	20.0

Appendix 3 Two rounds of experts consultation on nutrition management program items and content selection

Item	Deleted content	Add content	Modified contents
Management team	**Round 1:**<br>Rehabilitation therapist	**Round 1:**<br>Family members of older adults.<br>**Round 2:**<br>Informed consent was obtained from older adults and their family members.	**Round 1:**<br>1."Cafeteria staff" is changed to "meal service staff".<br>2.Modify the inclusion criteria and responsibilities of team member responsibilities.<br>**Round 2:**<br>Aged care workers should be included in the management standards.
Nutrition screening	**Round 1:**<br>Person in charge	None.	**Round 1:**<br>1."Nutrition screening" was revised to "nutrition risk screening".<br>2.Modification of screening requirements;<br>3."Result processing" was changed to "classification management after screening", and improve its content.
Nutrition assessment	**Round 1:**<br>Person in charge	**Round 1:**<br>1.Assessment of swallowing function: simple swallowing was used older adults were assessed by the Pharyngeal Handicap Questionnaire (EAT-10) swallowing function in young adults.<br>2.Assessment of nutrition and health status: increased tooth loss, denture wear, BMI, calf circumference, etc.	**Round 1:**<br>1."Nutritional assessment" was changed to "nutrition assessment".<br>2.Modification of assessment requirements.<br>3."Result processing" was changed to "classification management after evaluation", and improve its content.

Appendix 3 (Continued)

Item	Deleted content	Add content	Modified contents
Classification management	**Round 1:**<br>Person in charge	**Round 1:**<br>1.Risk factors for malnutrition.<br>2.Content of individual nutrition counseling;<br>3.Treatment of inadequate dietary intake.<br>**Round 2:**<br>Nutrition education should be conducted to increase the frequency of nutrition lectures and training.	**Round 1:**<br>1. "Classification management" is revised to "Development and implementation of Classification management program".<br>2. Modify and improve the content and precautions of nutrition education.<br>3. Change the frequency of individual nutrition counseling.<br>4. Modify and improve the content of meal support.<br>5. Modification and improvement of oral nutritional supplements and enteral nutrition. Application conditions, methods, precautions and effect evaluation content. "Evaluation time" and "evaluation method" were combined And "evaluation index and time", and modify its content.<br>**Round 2:**<br>1. Combine the first two items of "Individual nutrition counseling".<br>2. "Special dietary support" is included in nutritional support.<br>3. Incorporate "Appropriate Body Weight for Older Adults" into the nutrition assessment.<br>4. Changed "meal support" to "meal assistance.
Nutrition monitoring and effect evaluation	**Round 1:**<br>Person in charge	None.	None.

Appendix 4 Nutrition management program for older adults in nursing homes

**I. Nutrition Management Team Members and Responsibilities**		
Item		Contents
I-A Team members	I-A-1 Dietitian	Licensed dietitian or registered dietitian nutritionist; ≥ 5 years of related practice.
	I-A-2 Physician	Qualified as a licensed physician and practiced in medical and health care institutions for ≥ 5 years after registration; received training in nutrition management.
	I-A-3 Nurse	Registered nurse with a nurse's qualification certificate; practiced geriatric nursing for 5 years; received nutrition management training.
	I-A-4 Geriatric carers	Professional qualification certificate of senior care worker level 3 or above; ≥ 7 years of nursing experience; received nutrition management training.
	I-A-5 Catering staff	Qualified and have a health certificate; have catering skills.
	I-A-6 Elderly family members	Guardian or caregiver of an elderly person.
I-B Division of duties	I-B-1 Dietician	Admission assessment and regular assessment of nutrition; investigation and analysis of meals for older adults and improvement suggestions; individualized recipes or nutritional meals according to the preferences and needs of older adults; nutrition intervention plans; nutrition management and diet guidance for chronic diseases; nutrition monitoring and effect evaluation indicators.
	I-B-2 Physician	Assist dietitians in nutrition assessment, development of nutrition intervention programs, and management of adverse outcomes of nutrition intervention programs.
	I-B-3 Nurse	Establish nutrition management files for older adults; conduct nutrition risk screening; assist dietitians and physicians in nutrition assessment and formulation of a nutrition intervention plan; guide caregivers in implementation of the nutrition intervention plan; assist dietitians in nutrition monitoring and evaluation of impact; follow up on nutrition of referred older adults.
	I-B-4 Geriatric carers	Offer meal support, such as helping older adults with eating difficulties complete food and water supplies, observing and reporting the amount of food and surplus of older adults, assisting medical staff in tube feeding, and assisting nurses in nutrition monitoring, such as measurement of height and weight.
	I-B-5 Catering staff	Catering according to dietary doctor’s orders; food processing and production; daily ordering and delivery of meals; ensuring food safety, etc.

Appendix 4 (Continued)

**I. Nutrition Management Team Members and Responsibilities**		
Item		Contents
	I-B-6<br>Elderly family members	Participate in the formulation of a nutrition intervention plan for older adults; provide home diet care for older adults on weekends and holidays; and provide financial and emotional support for older adults.
**II. Aims of nutrition management for older adults in nursing homes**		
Item		Contents
Admittance criterion		Age ≥ 60 years old; older adults and their families are informed and consenting.
**III. Contents of nutrition management for older adults in nursing homes**		
Item		Contents
III-A<br>Nutrition risk screening	III-A-1 Screening Requirements	Completion of nutritional risk screening and establishment of nutritional management files for seniors during nursing-level assessments prior to admission to nursing homes.
	III-A-2 Screening instruments	Micro-Nutrition Assessment Scale (MNA-SF).
	III-A-3 Classification management after screening	● Normal nutrition: at least 1 intensive nutrition education per month and nutrition screening every 3–6 months.<br>● There is no risk of malnutrition, but risk factors for malnutrition such as oral and dental disease, dysphagia, mental disorders, dementia, long-term medication, dietary restrictions, or nutrition-related diseases include: actively correct risk factors for malnutrition. On the basis of individualized nutritional counseling. Repeat nutritional risk screening one month later.<br>● Malnutrition and nutritional risk: professional nutritional assessment by dietitians and physicians.
III-B Nutrition assessment	III-B-1 Assessment requirements	Assessment of the nutrition of individuals with malnutrition risk and malnutrition; assess the physical condition and condition changes to ensure their safety.

Appendix 4 (Continued)

**III. Contents of nutrition management for older adults in nursing homes**		
Item		Contents
III-B Nutrition assessment	III-B-2 Assessment contents	● Nutrition and health status: (a) Individual status: gender, age, tooth defect, denture wear, etc. (b) Disease condition: disease nature, species number, process and drug taking, etc. (c) Diet status: recent changes in food intake and individual nutritional needs, etc. (d) Laboratory examination: serum albumin, total protein, prealbumin, urinary creatinine, and total lymphocytes, etc. (e) Anthropometric measures: height, weight, handgrip strength, waist circumference, abdominal circumference, hip circumference, calf circumference, and triceps skinfold thickness. Weight changes and BMI were calculated from height and weight (<18.5 is thin, 18.5-23.9 is normal weight, ≥24 is overweight, ≥28 is obese). The most appropriate BMI for older adults is 21.0-26.9..<br>● Dietary assessment: food diversity and intake, dietary type and quality, food frequency, no restricted diet, etc.<br>● Eating habits: diet structure, satiety degree, diet taste, meals, whether picky, whether exercise is regular or quantitative, etc.<br>● Swallowing function: easy swallowing disorder questionnaire (EAT-10).
III-B Nutrition assessment	III-B-3 Classification management after assessment	Normal nutrition: at least 1 intensive nutrition education per month and nutrition screening every 3–6 months.<br>Simple malnutrition: according to the degree of malnutrition, develop a targeted nutritional management program, including correcting the risk factors for malnutrition, meal support, and nutrition education.<br>Malnutrition combined with acute or chronic diseases: develop an individualized nutrition management program, such as individualized nutrition counseling and nutrition support, and conduct nutrition education. Take them to the hospital if necessary, and nurses should follow up on the nutrition of older adults within a week after seeing the doctor and fill out the medical follow-up record sheet.<br>Overweight or obesity: develop an individualized weight management program.

Appendix 4 (Continued)

**III. Contents of nutrition management for older adults in nursing homes**			
Item			Contents
III-C<br>Formulation and implementation of classification management program	III-C-1<br>Identifies and corrects risk factors for malnutrition		● Determine malnutrition risk factors: (a) physiological factors: age and gender; (b) psychological factors: anxiety and depression; (c) disease factors: chronic wasting diseases, chewing or swallowing dysfunction, gastrointestinal dysfunction; (d) social factors: education, marital status, economic status, etc.<br>● Correct malnutrition risk factors: (a) Avoid unhealthy diets such as dieting, overeating, and partial eating; (b) Oral and dental care; (c) Assessment of medication and diet appropriateness; (d) Treatment and management of nutrition-related diseases; (e) Physical activity.
	III-C-2<br>Nutrition education	III-C-2-1<br>older adults	● Contents: basic knowledge of nutrition, the relationship between nutrition and health, the harm of bad eating habits, the formation of good eating habits, the way to correct an unreasonable diet, and self-monitoring of nutrition.<br>● Forms: (a) nutrition publicity materials, such as playing audio-visual materials and distributing nutrition manuals; (b) nutrition publicity display column; (c) public nutrition consultation activities; (d) nutrition knowledge lectures (held at least once every 2 months); (e) individual nutrition education.
		III-C-2-2<br>Nurse and geriatric carers	● Contents: nutrition knowledge such as recommended daily intake of food, nutrients, etc.; nutritional guidelines and individualized nutritional requirements; calculation of daily calorie requirements; assessment of daily food intake; configuration and use of nutritional preparations; use of nutritional screening and assessment tools and judgement of the results; training on nutritional management contents and procedures.
		III-C-2-3<br>Catering staff	● Contents: healthy cooking techniques, food ration (raw-cooked ratio of food), similar replacement of meat, eggs, and soy foods; dietary characteristics and cooking methods for diabetes, kidney disease, and other diseases; nutritious diet production standards; food storage and transportation.

Appendix 4 (Continued)

**III. Contents of nutrition management for older adults in nursing homes**			
Item			Contents
III-C Formulation and implementation of classification management program	III-C-2 Nutrition education	III-C-2-3 Catering staff	● Forms: (a) Nutrition knowledge lectures (held at least once every 2 months); (b) Meal preparation training: watching meal preparation videos, food molds, meal rationing tools, meal preparation training, visiting and studying in hospitals that carry out therapeutic diets, or inviting experienced personnel from outside mentoring (held at least once every six months).
		III-C-2-4 Family members of older adults	● Contents: basic knowledge of nutrition, home-based dietary guidance for older adults during holidays and weekends, the relationship between nutrition and health, and the importance of reasonable nutrition for older adults.<br>● Forms: (a) nutrition publicity materials such as nutrition manuals; (b) online push (WeChat group or public account); (c) public nutrition consultation activities.
		III-C-2-5 Cautions	● Different educational methods and contents should be selected according to the educational level and nutritional knowledge level of different educational objects.<br>● Adopt a combination of popular and individualized nutrition education methods, pay attention to the use of communication skills, and create a good learning environment and atmosphere.
	III-C-3 Individualized nutrition counseling	III-C-3-1 Forms	Face-to-face consultation, telephone, WeChat consultation.
		III-C-3-2 Contents	● Counseling and guidance on nutrition knowledge, healthy eating skills, and chronic disease management.<br>● Develop an individualized meal plan or recipe based on the nutritional and health status of older adults.
		III-C-3-3 Frequency	Individualized nutrition counseling as needed and monthly follow-up.

Appendix 4 (Continued)

**III. Contents of nutrition management for older adults in nursing homes**			
Item			Contents
III-C Formulation and implementation of classification management program	III-C-4 Meal support	III-C-4-1 Assisted meal	● Help with meals: provide meal delivery services for older adults in need of self-care, help, and care; provide accompanying meals or feeding according to older adult's self-care ability, cognitive status and swallowing function.<br>● Meal preparation: (a) Pay attention to nutrition and make reasonable meals, and make general food, soft food, liquid food and other special diets according to the needs of older adults or the doctor's advice; (b) Adjust the recipe once a week, inform older adults in advance and file them; (c) According to the eating habits of different elderly people make individualized diets according to cultural needs; (d) Determine energy needs according to the height, weight, and condition of older adults, and divide them into A/B/C packages according to energy levels.<br>● Diversified dietary supply: (a) Increase the supply of vegetables, fruits, milk and its products, coarse grains, fish, shrimp, and nuts to provide diversified meals. Eating more frequent, smaller meals and add fruit or milk as an extra meal in the recipe. (b) Nutritional meals should be varied, with reasonable proportions of protein, fat, carbohydrates, vitamins, and minerals to achieve balanced nutrition.
		III-C-4-2 Environmental support	● Create a harmonious and comfortable dining environment: encourage older adults to arrange meals independently, fixed table meals.<br>● Accompanying meals: encourage seniors to eat together in groups.
		III-C-4-3 Meal frequency and time	● Except for older adults who have special requirements such as the need for small and frequent meals. A small amount of fruit or snacks can be eaten before or between meals three times a day to increase the frequency of eating.<br>● Eat on time and provide ample meal time.

Appendix 4 (Continued)

**III. Contents of nutrition management for older adults in nursing homes**				
Item			Contents	
III-C Formulation and implementation of classification management program	III-C-4 Meal support	III-C-4-4 Result processing	● Continue the diet plan if no more than 1/3 is left after meals.<br>● If the amount of food remaining exceeds 1/3, the nursing staff should inform the nurse of the remaining amount. The nurse will evaluate the rationality of the diet plan for older adults and the impeding factors that affect older adult's eating, and cooperate with the nutritionist to formulate a nutrition management program. For example, correction of meal-impeding factors, health education or adjustment of diet plan, and nutritional support such as nutritionally fortified foods and oral nutritional supplements can be added if necessary.	
	III-C-5 Meal support	III-C-5-1 Goals of nutritional support	The recommended nutritional support energy intake is 25-30kcal/(kg-d), and the protein intake is 1.0-1.5g/(kg-d), and individual adjustment is made according to the nutritional needs of different elderly people and people with different disease conditions.	
		III-C-5-2 Nutrition support stage	Stage 1: fortified foods	● Applicable conditions: oral intake but insufficient intake or energy intake below 50% of requirement for more than 1 week.<br>● Methods: choose appropriate nutrition preparations according to older adult's nutrition, disease status, gastrointestinal function, renal function, infusion route and economic situation.

Appendix 4 (Continued)

III. Contents of nutrition management for older adults in nursing homes				
Item			Contents	
III-C Formulation and implementation of classification management program	III-C-5 Meal support	III-C-5-2 Nutrition support stage	Stage 2: Oral Nutritional Supplements (ONS)	● Cautions: (a) Adjust the type, taste, texture, and timing of meals according to the taste and eating ability of older adults. (b) Pay attention to oral care to prevent the occurrence of complications such as aspiration pneumonia. (c)The determination of the intake of energy and protein and other nutrients should be based on the age of older adults, basal metabolic rate, activity level, etc., and adjustments should be made through regular monitoring. (d) Improve the compliance of older adults by adjusting the taste of the preparation and psychological care.<br>● Effect evaluation: the efficacy and tolerability of ONS should be assessed weekly.
		III-C-5-2 Nutrition support stage	Stage 3: Enteral Nutrition (EN)	● Applicable conditions: there is malnutrition or nutrition frisk, and the gastrointestinal tract function is normal or basically normal, but the ONS still cannot meet 60% of the target energy demand for 3 to 5 days or can not eat orally.<br>● Methods: the method should be selected according to the caregiver's ability to care, the activity of older adults, the disease status and the cost. Patients with active and normal gastrointestinal function should be given regular bolus injection, and those with risk of aspiration and gastrointestinal dysfunction should be instilled by gravity.

Appendix 4 (Continued)

III. Contents of nutrition management for older adults in nursing homes

Item		Contents		
III-C Formulation and implementation of classification management program	III-C-5 Meal support	III-C-5-2 Nutrition support stage	Stage 3: Enteral Nutrition (EN)	● Cautions: (a) Standard whole protein formula is appropriate for most elderly EN; long-term use of optimized fatty acid formula can improve lipid metabolism and reduce cardiovascular events. (b) Dietary fiber intake should be ⩾ 25g/d to prevent constipation. (c) Unused nutrient solution should be stored in the refrigerator at 0°C-4°C and the shelf life is 24h. (d) The speed of nasal feeding should not be too fast, once every 2h, 200ml at a time. Flush the tube with a little warm water before and after each feeding. (e) Raise the head of bed 30~40° before each nasal feeding 30~40°, and keep the height for 20 minutes after nasal feeding to prevent reflux.<br>● Effect evaluation: the nutrition, gastrointestinal function, tolerance, and complications of older adults should be assessed once a week.
	III-C-6 Weight management	● Overweight: dietary weight loss should be avoided to prevent loss of muscle mass and concomitant functional decline; weight loss management should be individualized, requiring assessment of the impact of excess weight on quality of life and consideration of the need for regular exercise.<br>● Obese: Moderate energy restriction and slow weight loss; energy should be limited moderately and weight should be lost slowly. The diet and exercise plan should be based on physiological characteristics and physical conditions, and real-time monitoring and recording of food, water intake and exercise volume.		
III-D Nutrition monitoring and effect evaluation	III-D-1 Metrics and frequency of evaluation	● Anthropometric indicators: height, weight, waist circumference, hip circumference, calf circumference and skin fold thickness were measured once a week. Calculate BMI based on height and weight.<br>● Laboratory test indicators: serum albumin, total protein and prealbumin, etc. The frequency of the test shall be determined by the physician or nutritionist according to the actual situation.<br>● Reassessment of nutrition: food intake 24 hours a day for 3 days should be assessed monthly.		

Appendix 4 (Continued)

**III. Contents of nutrition management for older adults in nursing homes**		
Item		Contents
	III-D-1 Result processing	● When the intake of older adults is 60% of the target energy requirement for 3-5 consecutive days, the nutrition has improved, and the relevant nutritional knowledge has been mastered, the nutritional management should be stopped. The nutrition should be assessed once a month, and periodically monitor every 3--6 months after 3 months.<br>● When the intake is less than 60 % of the target energy requirement for 3-5 consecutive days, the nutrition has not improved, the nutrition-related knowledge has not been mastered, and there is a nutritional-related disease. Assess whether it is necessary to change the eating pattern, and whether to change or continue the nutritional management program. Nutrition monitoring and evaluation should be repeated within a month.
